# Towards Automated Three-Dimensional Tracking of Nephrons through Stacked Histological Image Sets

**DOI:** 10.1155/2015/545809

**Published:** 2015-06-15

**Authors:** Charita Bhikha, Arne Andreasen, Erik I. Christensen, Robyn F. R. Letts, Adam Pantanowitz, David M. Rubin, Jesper S. Thomsen, Xiao-Yue Zhai

**Affiliations:** ^1^Biomedical Engineering Research Group, School of Electrical & Information Engineering, University of the Witwatersrand Johannesburg, Private Bag 3, Johannesburg 2050, South Africa; ^2^Department of Biomedicine, University of Aarhus, 8000 Aarhus C, Denmark; ^3^Department of Histology and Embryology, China Medical University, Shenyang, Liaoning 110122, China

## Abstract

An automated approach for tracking individual nephrons through three-dimensional histological image sets of mouse and rat kidneys is presented. In a previous study, the available images were tracked manually through the image sets in order to explore renal microarchitecture. The purpose of the current research is to reduce the time and effort required to manually trace nephrons by creating an automated, intelligent system as a standard tool for such datasets. The algorithm is robust enough to isolate closely packed nephrons and track their convoluted paths despite a number of nonideal, interfering conditions such as local image distortions, artefacts, and interstitial tissue interference. The system comprises image preprocessing, feature extraction, and a custom graph-based tracking algorithm, which is validated by a rule base and a machine learning algorithm. A study of a selection of automatically tracked nephrons, when compared with manual tracking, yields a 95% tracking accuracy for structures in the cortex, while those in the medulla have lower accuracy due to narrower diameter and higher density. Limited manual intervention is introduced to improve tracking, enabling full nephron paths to be obtained with an average of 17 manual corrections per mouse nephron and 58 manual corrections per rat nephron.

## 1. Introduction

The kidney performs the vital functions of water and solute transport, blood pressure regulation, and urine concentration through the functional unit of the nephron. The microarchitecture of the kidney has recently been the focus of a number of studies [1–3]. In particular, the functional implications of the renal microstructure on the underlying mechanisms involved are of great interest [4–6]. A deeper characterisation of the microarchitecture enables the development of models to accurately simulate the functionality of the kidney. Some important data includes the ratio of short- to long-looped nephrons, relative length, type, and distribution of parts of the nephron.

A large database of histological images of mouse [7] and rat [8] kidneys was made available from previous studies performed at the Aarhus University, Denmark. The previous work involved manual tracking of the paths taken by a few hundred nephrons through the image sets and thereafter performing an in-depth analysis of the findings.

The ultimate objective of this study is to improve understanding of the architecture of the human kidney; however, tracking of human nephrons is subject to a number of practical limitations and has been left for future work. It is anticipated that several structural and functional aspects of mammalian kidneys, including human kidneys, may be elucidated through these studies of rodent histology.

Each mouse and rat dataset comprises, on average, 1000 and 3000 images, respectively. Manually tracking one long-looped mouse nephron requires tracking about 1800 elements, which takes hours to carry out. The extensive time and effort required for such datasets make it impractical to track large numbers of nephrons. Therefore any semi- or fully automated tracking procedure would be beneficial.

This created the need for an automatic tracking algorithm which could potentially be used as a standard tool on multiple datasets. This would allow the renal characterisation of multiple species as well as pathological specimens. Since the microstructure of nephrons can vary in the same kidney, it is important to obtain large samples when taking measurements, such as lumen diameters and nephron lengths, in order to render the findings more statistically accurate and representative of a variety of kidney specimens.

It is important to note the difference between automatic tracking and segmentation. The latter is the isolation of independent structures in images, such as the separation of organs in computed tomography and magnetic resonance images [9, 10], or the differentiation between tissue types in histological images, mostly for purposes of visualisation or further processing. In contrast, automatic tracking utilises the results of segmentation to create an abstract computational reconstruction of the structure for purposes of accurate measurement.

Currently, there exists no method for the automatic tracking of nephrons through serial slices. However, methods for automatic tracking of other biological structures do currently exist, with a common example being that of blood vessels in retinal images [11–13]. Other structures for which automatic tracking has been attempted include the dendrites of individual neurons and the portal and hepatic venous trees of the liver [14].

However, the methods from the aforementioned applications cannot be directly applied to the nephron tracking problem due to a number of factors. A crucial difference is that there are hundreds to thousands of nephrons [15] that need to be independently tracked through serial slices (a three-dimensional problem) as opposed to one or a few structures in single images (a two-dimensional problem). In particular, the tortuosity of the nephrons poses a major challenge. Nevertheless, several concepts from existing tracking applications have been adopted in the current approach, such as graph-based tracking, metrics to indicate confidence per iteration, and a set of validation rules to reduce error.

This paper presents a methodology for automatically tracking nephrons through images obtained from serial kidney sections using image processing, feature extraction, graph-based tracking, and machine learning techniques. The combined application of these techniques presents a novel approach to the nephron tracking problem.

The research aims to determine how effectively and accurately an automated approach can be compared to the manual method and to quantify how much manual intervention is necessary in the automatic approach to track the paths of entire nephrons. Once tracked, the results can be processed to extract useful metrics and statistics.

## 2. Data Acquisition

The dataset was obtained from two previous projects performed at the University of Aarhus as described in the following.

**Experiment 1 .**

Kidneys from three 8-week-old male mice were fixed through the abdominal aorta with glutaraldehyde. The tissue blocks were cut perpendicular to the longitudinal axis from the surface of the kidney to the papilla. The tissue blocks were fixed overnight in the same fixative and postfixed with OsO_4_, en bloc stained with uranyl acetate, and embedded in flat molds in Epon. From each of the three mouse kidneys 897, 990, and 1064 2.5 *μ*m thick consecutive sections were obtained using a microtome equipped with a Diatome histoknife. The sections were stained with toluidine blue when heated onto the microscope slices [7].

**Experiment 2 .**

Kidneys from three 3-month-old male Wistar rats were cut into 4252, 4384, and 4541 2.5-*μ*m thick serial sections and processed as described above [8]. All animal experiments were carried out in accordance with provisions for the animal care license provided by the Danish National Animal Experiments Inspectorate.

The multiple serial sections were digitized using a microscope equipped with a digital camera attached to a standard PC. In Experiment 1, the sections were digitized into images using a ×4 objective lens resulting in a final image size of 2500 × 1675 pixels and an isotropic pixel size of 1.16 *μ*m. In Experiment 2, the images were recorded using a ×3 objective lens, producing images of 2750 × 2500 pixels with an isotropic pixel size of 1.550 *μ*m. The multiple digitized serial images were subjected to a classic rigid registration followed by a nonrigid transformation using custom-made software written in C [16–18].

## 3. System Overview

**Figure 1:**
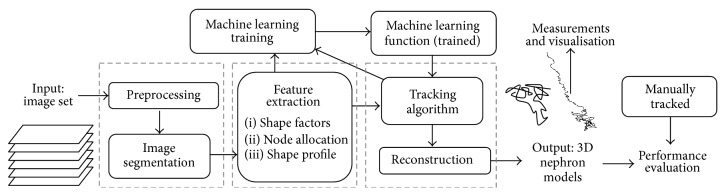
A high level overview of the nephron tracking system, showing the main subsystems and the flow of information between them.

From a methodological perspective, a tracking problem would fit the generic architecture of a Computer Aided Diagnosis (CAD) system [18] with stages of preprocessing, defining regions of interest, feature extraction and selection, and classification [19].  Figure 1 describes the architecture of the tracking system developed in the present study.

The system was implemented in MATLAB [20] as a series of independent modules where structures of information are progressively passed from one stage to the next. This framework is related to an object-orientated approach in that the major functions are decomposed into independent, reusable blocks. The development of the system is incremental, involving continuous reiteration through the three main stages to achieve optimal performance.

## 4. Image Preprocessing

The purpose of the preprocessing stage is to prepare the images for the feature extraction stage, by creating uniformity among all nephron cross sections and addressing nonideal factors. The images are processed such that required features (nephron cross sections) are enhanced while unwanted features (such as interstitial tissue cross sections, large blood vessels, background pixels, and large artefacts) are filtered out or reduced.

The lumens of the nephrons are the object chosen to be isolated because they are more easily and accurately isolated than the nephron walls which touch each other. Each image undergoes the following.

- Conversion to grayscale is performed as the staining used on the specimens (toluidine blue [7]) results in all structures being monochrome. If a more differentiating staining method was to be used in future image sets, the colour information should be retained.
- Background removal is achieved by forming a background mask through a threshold filtration, large component extraction, and morphological image closing using a circular kernel. The mask is then inverted and applied to the original image by multiplication.
- Histogram equalisation is performed in order to counteract uneven intensities which are commonly present. Global and local adaptive equalisations are applied through the use of a large and small equalisation window, respectively [21].
- Simple thresholding creates a binary image. The threshold value is chosen so that it does not allow independent lumens to merge while also not letting small nephron cross sections disappear.
- Morphological erode/dilate cycles result in the removal of thin interstitial tissue cross sections. The kernel is chosen carefully so as to not mistakenly remove small nephron cross sections.
- Binary components that are very small (<10 pixels) and very large (>100 000 pixels) can be confidently identified to not be nephron cross sections and are removed.

Obtaining this final binary image is one of the most important tasks, as the accuracy of the following stages depends on how well the cross sections are isolated from one another. Many parameter values are critical when deciding on how many interstitial tissue cross sections appear in the images. A compromise must be made between the number of interstitial tissue cross sections present and the number of small nephron cross sections that do not get eliminated.

Further preprocessing involves the removal of highly distorted images and replacing them with the image above or below (so as to not have missing image numbers in the set). An average of 4 images per dataset has been manually replaced. However, an automatic method can be devised if a larger number of images are defective, for example, analysing the mean intensity of each image in the image set.

### 4.1. Sigmoid Function for Automatic Parameter Variation

A transition zone in the outer medulla exists where the thick descending limb (≈60 *μ*m in diameter) suddenly narrows to a diameter of 10–15 *μ*m to form the thin descending limb [22, 23]. This change requires almost all parameters of the preprocessing steps to change to ensure that nephron cross sections of all sizes are extracted. In order to automatically accommodate this change in morphology, the parameters of the preprocessing steps are made to vary according to a modified sigmoid function [24] which has its inflection point set at the transition zone. This also allows relatively constant parameter values in the cortex and inner medulla. The parameters of the sigmoid functions must be manually chosen through experimentation as part of system calibration.

## 5. Feature Extraction

Feature extraction aims to simplify and concentrate useful information from raw data. Within the images, large amounts of the data are not useful, for example, the large number of pixels making up the background. The pixel information can instead be condensed into a set of features per nephron cross section, which represent the problem to a sufficient degree. Intuitively, the most useful information about a single nephron cross section is its size, shape, colour, and location.

### 5.1. Image Segmentation

Connected component segmentation [21] (4-connected neighbourhood) is used to segment the image into independent nephron cross sections. Watershed segmentation is another possible segmentation technique, which could perform better in cases where independent lumens incorrectly merge through a few connected pixels. However, this method tends to oversegment the image [25], resulting in the division of elongated nephron cross sections.

### 5.2. Node Allocation

A* node* is defined as a point coordinate in the three-dimensional (3D) image space. The pixel locations per nephron cross section can be reduced into a set of nodes allocated along the cross section (e.g., a circular nephron can be represented by one centre coordinate, instead of hundreds of pixel locations). An elongated cross section can have multiple nodes along its length. This abstraction greatly simplifies the problem, reduces the size of the data, decreases the computational load on subsequent stages, and concentrates the significant information.

*K*-means clustering is used to allocate nodes [26]. Each nonzero pixel on a single isolated binary cross section is designated as an observation. If the nephron cross section is circular or small, one centroid is requested (*K* = 1). For elongated nephron cross sections, the *K* value increases until the mean distance between adjacent nodes is less than a desired value. This ensures an adequate number of nodes are allocated per nephron cross section depending on its size.

### 5.3. Shape Measurements

**Figure 2:**
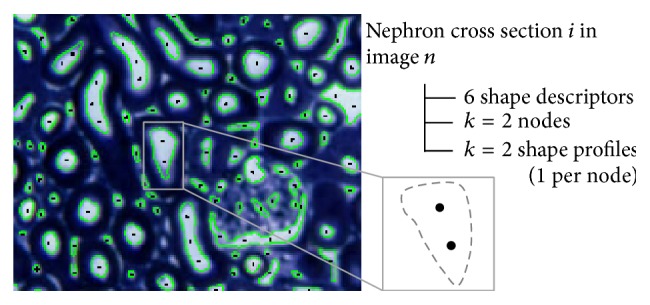
An example of a raw image is shown. The extracted binary cross sections after preprocessing are highlighted in green and the allocated nodes are shown as black dots. Each cross section will have *k* nodes, 6 shape factors, and *k* shape profiles. Many cross sections in the cortex are not of actual nephrons but rather of the interstitial tissue between them. The glomeruli are also highly segmented.

Tracking of a nephron using only the 3D set of nodes results in the linkage of multiple nephrons, blood vessels, and interstitial tissue. By only considering the point cloud, the algorithm is blind to a large amount of available information. Therefore, shape information of each cross section is also captured. Each node gets assigned a group of shape metrics and a shape profile as shown in Figure 2. The idea behind incorporating shape information into the tracking is to make the algorithm intelligent and highly confident at each incremental step of the process.

#### 5.3.1. Shape Factors

A shape factor refers to a dimensionless value that is dependent on an object's shape but is independent of its size [27]. These metrics are calculated using various measurements of an object, such as its area, perimeter, and diameter. They usually indicate the degree to which an object deviates from an ideal shape, such as a square or circle [27]. Shape factors are extracted to capture abstract information about each cross section along with the nodes. Circularity, eccentricity, solidity, and aspect ratio were chosen as useful descriptors for the cross sections. Area and minor axis length are also captured as absolute-valued descriptors.

#### 5.3.2. Shape Profile

The shape factors are useful for cross sections that are round and elliptical, but they do not adequately describe cross sections that are more arbitrarily shaped, such as glomeruli or interstitial tissue cross sections. As an additional feature, the shape profile, or centroidal profile, of each cross section is calculated.

The shape profile of an object is a polar plot of the distance to its boundaries with respect to a reference point [21]. It transforms a two-dimensional shape representation into a one-dimensional plot [21]. The centroid is commonly selected [21], but the nodes allocated in the previous step have been chosen instead as they are more relevant to the problem and will allow an accurate relative comparison of shape profiles between nodes.

First, the edges of a single cross section are obtained using a Sobel edge detector [28]. This method produces a well-defined closed curve around the cross section. The edge pixels are then processed into an ordered set of points. The angles and radii relative to the reference point are calculated as in

(1) θ=arctan⁡Pedgey−PrefyPedgex−Prefx,rθ=Pedge−Pref,

where **P**_**edge**_ is the vector of edge coordinates, *P*_ref_ is the reference coordinate, ***θ*** is the vector of angles, and **r**(*θ*) is the vector of radial distances. The shape profile undergoes unwinding and interpolation at desired angles in order to eliminate multivalued points and produce a consistent feature set. The degree of abstraction is dependent on the angle increment [21], which was chosen to be 15°.

## 6. Tracking Algorithm

When a nephron is manually tracked by the eye, an intuitive process is used by the brain. Once a single nephron cross section has been fixated, a nephron cross section within the same vicinity is searched for in the next image. Size, shape, and colour are also subconsciously compared. The tracking algorithm uses a similar process, with a number of generalised rules to accommodate the tortuous path taken by the many nephrons. The algorithm is highly dependent on the quality of preprocessing and the accuracy of feature extraction stages.

A graph-based approach similar to algorithms like the A-star search algorithm is employed for tracking [29]. The algorithm forms a graph in 3D space by establishing edges between the nodes previously allocated during feature extraction. Open and closed lists are used to manage the unexplored and explored nodes, respectively. Each node is stored along with its parent node, forming a linked list. Ideally, given a starting seed, edges should be formed such that all nodes belonging to one nephron are collected in the closed list. Prior to proceeding, a few symbols are defined:

$I_{n}$: image *n*,
${𝒞}_{n}$: the set of all nodes in image *n*,
$c_{n}^{i}$: the set of nodes on cross section *i* in image *n*,
${c_{k}}_{n}^{i}$: the *k*th node on cross section *i* in image *n*.

### 6.1. Edge Formation

**Figure 3:**
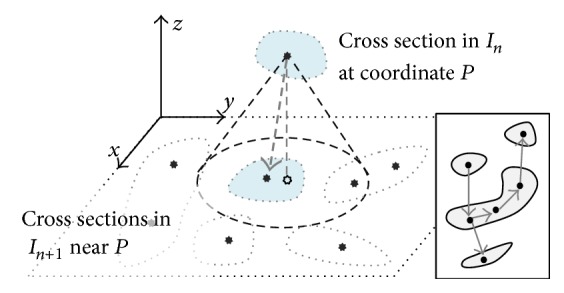
Each node in image *n* has the potential to connect to 2 nodes vertically (in images *n* + 1 and *n* − 1) within some tracking radius and 1 node horizontally on the same cross section as itself. This allows cross sections to be linked through turns and bends.

The edges are established through a controlled set of criteria. Given a particular node *c*_*k*__*n*_^*i*^  in image *I*_*n*_, it has the potential to connect to three other nodes through two types of edges as shown in Figure 3:

(2) $$\begin{array}{c} \min\left(\;\left\|\;C_{n\pm 1}-{c_{k}}_{n}^{i}\;\right\|<r_{\text{track}}\;\right). \end{array}$$

#### 6.1.1. Vertical Edge

It includes potential connections to cross sections in the image above (*I*_*n*−1_) and below (*I*_*n*+1_) the current cross section. Nodes are searched for which lie within some tracking radius around the current node; that is, a node satisfying the following condition will become a child node of the current node.

Only one node is allowed to be formed in each direction. If multiple nodes satisfy the condition, the one with the smallest Euclidean distance is used. The confidence of a vertical edge is <1, as the possibility of linking to an incorrect cross section exists due to the large number of closely packed nephrons.

#### 6.1.2. Horizontal Edge

It involves linking all nodes that lie on the same cross section as the current node, that is, *c*_*n*_^*i*^. The current node is termed the “entering” node. The pairwise Euclidean distances between all nodes are used to establish the linkage between the nodes.

### 6.2. Local Image Registration

Local alignment is needed (in addition to the alignment in the previous study [8]) due to the presence of local image distortions and progressive change in morphology. Images *I*_*n*_, *I*_*n*+1_, and *I*_*n*−1_ are cropped around the current node location. The subimages in *I*_*n*+1_ and *I*_*n*−1_ are cross-correlated against *I*_*n*_ in order to obtain the translational *x*- and *y*-offset between the images [30]. These are typically only a few pixels but have a large impact on the accuracy of tracking since some nephron cross sections are also just a few pixels wide. This local alignment only takes translation into account; it is assumed that local rotational offsets are minimal. Future work could explore the increase in accuracy obtained with the use of more complex image registration methods such as a nonrigid transform. Once a link has been made between cross sections, the transformation is reversed to avoid accumulation of the offsets.

### 6.3. Skipping Images

An image may be termed defective if it has a large number of interfering artefacts or distortions, which obscure cross sections of the nephron at hand. These images can in general be skipped while tracking the nephron. However, a maximum of 2 images (the equivalent of 5 *μ*m of the specimen) may be skipped at a time, as the morphology can change vastly in this span and would introduce too large a probability of error in tracking (e.g., jumping onto another nephron). A set of skipping criteria are established using a direction buffer and refractory period to prevent skip attempts from occurring too frequently (from every dead end).

### 6.4. Validation Steps

The steps discussed thus far would work if the data only contained information of the nephron cross sections. However, many of the cross sections actually belong to interstitial tissue and blood vessels which are randomly dispersed between the nephron cross sections and lie in close proximity to the nephron at hand. Even though the correct nephron path may be found, much interference is caused by interstitial tissue cross sections, potentially causing the path to branch from the nephron's path and even link onto other nephrons. A rule base of three validation steps is incorporated into the tracking algorithm in order to eliminate incorrect moves from one cross section to another.

- *Distance Validation*. The Euclidean distance (in the *x*-*y* plane) between a parent and potential child node must be less than the sum of their radii (half the minor axis length is used). This ensures that even if a cross section lies within the tracking radius, consistency in terms of size and relative displacement is maintained. Many cases of interstitial tissue cross sections linking to nephrons are eliminated by this rule.
- *Bidirectional Movement Validation*. If a move is made from node A in image *I*_*n*_ to node B in image *I*_*n*+1_, then an attempted move from B to image *I*_*n*_ must lead back to node A (i.e., bidirectionality must be maintained). If not, the move is discarded. Moves between interstitial tissue cross sections are typically not connected in this manner and are hence largely eliminated.
- *Skipping Validation*. This ensures that a move involving a skip is only allowed if the shape of the cross section remains relatively constant during the skip. This means that skips will not be allowed on turns and bends, as this presents a high chance of error. The change in shape is measured by the percentage change in the six shape factors.

### 6.5. Reconstruction

The path is reconstructed through inference of the parent-child node pairs. The longest path forms the nephron path, while shorter branches are eliminated as they are most likely ambiguous nephron paths or pieces of interstitial tissue that were mistakenly linked. Each coordinate can be linked to its shape factors, enabling a 3D rendering of the nephron path with a varying lumen radius.

Lastly, the automatically tracked path must be evaluated in 3D space. At this stage, known information about the problem can be used, for example, the proximal and distal convoluted tubules intertwine and must thus be in the same vicinity in the cortex [7], or the proximal convoluted tubule is longer and more convoluted than the distal [7]. Incorrect paths can be eliminated by comparison with typical 3D features of nephrons, such as curvatures of the bends. If the results do not adhere to one or more of these expectations, it could then be that the result is incorrect.

## 7. Validation Using Machine Learning

The validation rule base results in some nephrons being correctly tracked, while others are incorrectly linked to other nephrons, interstitial tissue cross sections, and blood vessel networks. A large amount of information has not yet been taken into account, such as the shape profile and shape metrics. The purpose of the machine learning (ML) stage is to incorporate some form of intelligent decision making when linking one node to another during tracking. This is done by assessing the shape descriptors and other features of the two cross sections through a trained classifier. A supervised Artificial Neural Network (ANN) and Support Vector Machine (SVM) have been used to classify a move from one cross section to another as either valid or invalid. This classification is used by the tracking algorithm to make decisions during tracking.

### 7.1. Feature Selection

The chosen features must fully characterise a move from one cross section to another and provide a good degree of distinction between different types of examples. Since two cross sections are being compared, it is useful to look at combined features. A total of 66 features are used including

- the means and differences between the shape factors,
- the Euclidean distance between nodes in the *x*-*y* plane,
- the *z* position of the nodes relative to the image set to indicate depth into the kidney, that is, cortex to medulla,
- the image difference, normally 1, that can be 2 or 3 if images have been skipped,
- image alignment offset, high offset coupled with other odd features, which may be a flag for an abnormal move,
- the shape profiles of the cross sections at 15° intervals and a correlation metric of the shape profiles.

### 7.2. The Training Process

**Figure 4:**
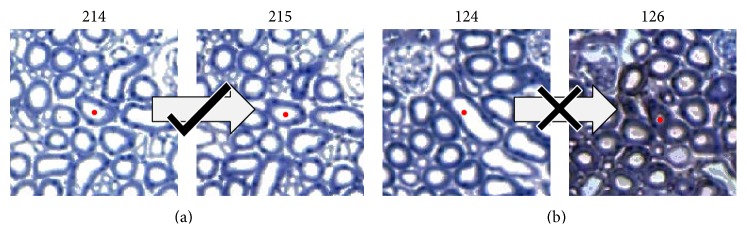
The moves attempted by the unregulated tracking algorithm are captured, displayed, and labelled to form training examples for the neural network. The image shows examples of a valid (a) and invalid (b) move, which will be labelled with a “1” and a “3,” respectively.

The training set is created by capturing moves (pairs of cross sections) during unsupervised tracking (without any machine learning validation) of a chosen set of nephrons. Each parent-child pair is assigned a label as in Figure 4.

Five output classes listed in Table 1 were chosen to form the output matrix. A voting scheme [31] between the classes is then used to determine the final classification as valid or invalid. Class 4 is used to terminate tracking at the glomerulus while class 5 is used as a “region signal” to change the mode of tracking between the cortex and inner medulla. The shape factors and descriptors belonging to each cross section in the pair can be extracted as required and the 66 features are then combined to form the input matrix. A multiclass classifier is produced using the one-versus-all approach [32].

In addition to manual selection of examples, a method involving a feedback process between the tracking algorithm and the training process is used in order to collect a fair number of examples per class. This prevents the formation of a skewed dataset or underrepresentation of a certain class, which may affect classification accuracy.

A threshold is applied to the continuous output of the ANN in order to deem the result positive or negative. This threshold has an impact on the sensitivity of invalid move rejection. For the SVM, the width of the radial basis function (RBF) kernel has the analogous effect. It is critical that false positives are minimised as these would halt the tracking process by blocking a valid move along the path of the nephron, hence preventing the rest of the nephron from being tracked. A false negative on the other hand would allow an incorrect path to be formed, but the incorrect path is typically halted due to the presence of many invalid moves through interstitial tissue and is therefore not as critical as a false positive.

## 8. Manual Intervention

**Figure 5:**
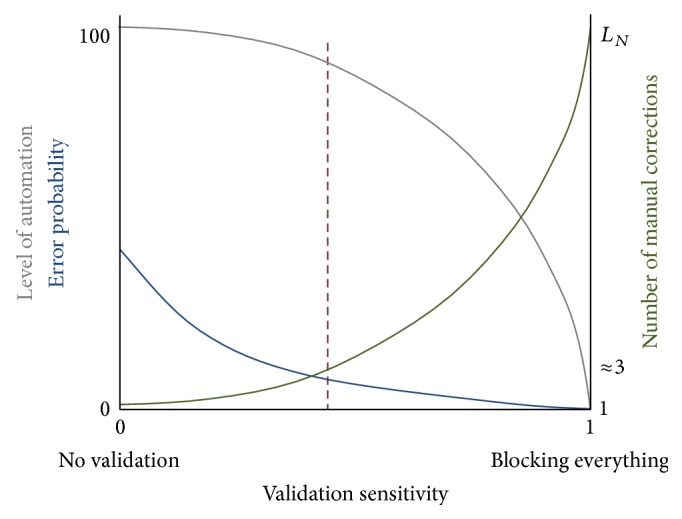
The number of false positives increases with increasing validation sensitivity, resulting in premature termination of tracking. This means only a portion of the nephron is tracked, but with a low error, where error refers to deviation onto an incorrect path. If manual correction is used, the number of corrections required for continuation of tracking will increase with sensitivity (up to *L*_*N*_, the length of the nephron). This means a decreased level of automation but also decreased chances of error. The graph is merely conceptual.

Premature termination of tracking (due to nonideal preprocessing, feature extraction, image artefacts, or distortions) commonly occurs in the inner medulla. Image spatial resolution is a limiting factor for these small cross sections. One way of overcoming premature termination without introducing an error is to allow the user to manually bypass problematic cross sections at the end points of the automatically tracked path. This, of course, reduces the automaticity of the system but still dramatically reduces the time and effort required for the manual tracking task. The degree of automation can be controlled by sensitivity of the validation stages, as shown in Figure 5.

## 9. Results

### 9.1. Automatically versus Manually Tracked Nephrons

The accuracy of an automatically tracked nephron is measured against the manually tracked data, which forms the gold standard. The following is defined for ease of description:

$\Upsilon_{n}$: the manually tracked path of nephron *n*,
$\Psi_{n}$: the automatically tracked path of nephron *n*.

When the result has a low degree of correctness, it is because either tracking terminated prematurely or the path deviates onto an incorrect one (linkage with another nephron, blood vessel, or interstitial tissue cross sections), or a combination of these. The outcome of the tracking of a particular nephron is hence evaluated using two correctness measures:

1. *α*_*n*_ = % of Ψ_*n*_ that is correct – “accuracy,”
2. *β*_*n*_ = % of *Υ*_*n*_, that Ψ_*n*_ covers – “extent.”

These are calculated using per image residuals between the automatic and manually tracked coordinates. *α* measures the similarity to the manually tracked nephron. It is low if the path deviates onto other structures and high if the tracked path contains data of only the target nephron, be it a small or large portion. *β* measures how much of the target nephron is tracked; it is low (relative to the ideal *β* value per segment) if only a small portion is tracked. It can still be high if the path branches onto incorrect structures, as long as a large part of the target nephron is found.

**Figure 6:**
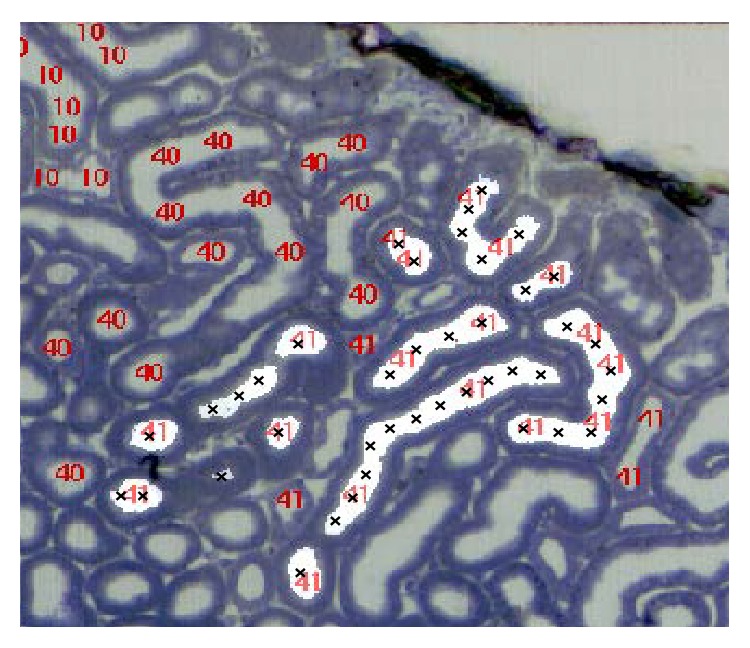
An example of a labelled image is shown with the red numbers representing the different manually tracked nephrons. The automatically tracked nephron (number 41) is superimposed, shown in white with black crosses at the nodes. Unlabelled “41” cross sections are of the DCT which was not tracked in this instance.

**Figure 7:**
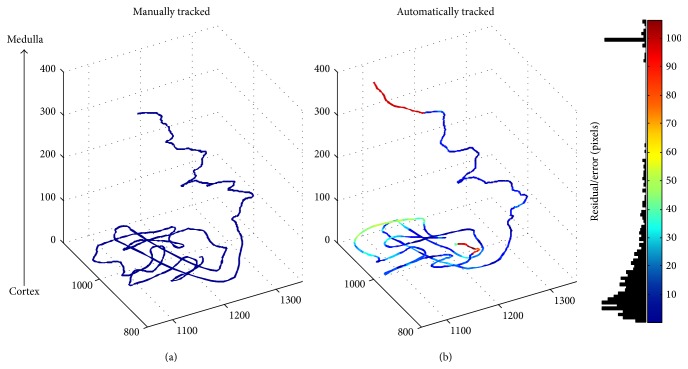
A manually tracked mouse nephron is shown on the left. The same nephron is successfully tracked automatically by the algorithm (with *α* = 97%) and is shown on the right. Tracking terminates automatically at the glomerulus. Note that, in each plot, the cortex is shown at the bottom and the DTL extends upwards. The path is coloured by the error, or residual, with respect to the manually tracked nephron. Slight discrepancies in appearance are due to different image alignments and different point coordinates used by the two methods. The distal DTL has greater error simply because the manual path was not tracked as far (therefore, *β* > 100%). It can be seen in the error histogram that most of the residuals are less than 15 pixels. The correct paths of the PCT, PST, and DTL are tracked.

The tracking algorithm successfully tracks large portions of the nephrons automatically, occasionally requiring manual intervention in order to obtain full nephron paths. 16 nephrons from 2 mouse datasets and 11 nephrons from 2 rat datasets were chosen to form a test set. These were not used to form the training set for the machine learning algorithms. Different parts of the nephrons were tracked with varying accuracies and extents as shown in Table 2, due to differing tubule characteristics. In particular, the proximal convoluted tubule (PCT) and proximal straight tubule (PST) were tracked well, while the descending thin limb (DTL) and ascending thin limb (ATL) of the loop of Henle were more problematic in both the mouse and rat datasets. Automatic tracking of the PCT of a rat nephron is shown in Figure 6 and example of the PCT, PST, and DTL of a nephron tracked both manually and automatically is compared in Figure 7. The thick ascending limb (TAL) is tracked well in both the mouse and rat while the distal convoluted tubule (DCT) is only tracked well in the rat due to its larger diameter.

Tracking a full mouse nephron requires an average of 19 manual corrections while a full rat nephron requires 58 manual corrections. The frequency of manual intervention is dependent upon the number of image artefacts and distortions encountered along the path of the nephron, as well as the visibility of the cross sections. A longer path (in terms of the number of moves) requires more corrections; for example, the rat nephrons are on average 4.7 times longer than mouse nephrons.

**Figure 8:**
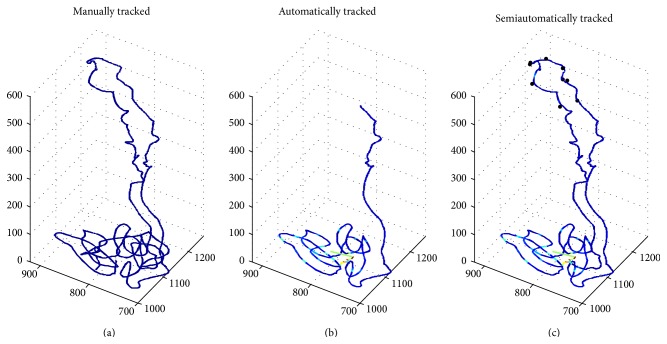
A manually tracked mouse nephron is shown on the left. The PCT and PST are successfully tracked automatically as shown in the middle plot. Tracking terminates due to diminishing tubule size coupled with artefacts in the inner medulla. A more complete nephron path is obtained with 5 manual corrections on the DTL and 4 on the ATL, as shown on the right plot (semiautomatically). The paths are coloured by the error, or residual, with respect to the manually tracked nephron. The maximum residual (shown as dark red) in this instance is 35 pixels. The black asterisks are points of manual correction. This is acceptable considering that a total of 1222 coordinates make up this path. *α*_AUTO_ = 97.13%; *β*_AUTO_ = 39.84%; *α*_SEMI-AUTO_ = 98.77%; *β*_SEMI-AUTO_ = 90.23%.

The average number of corrections required for each part of the nephron is contained in Table 2. Most corrections are for the DTL and ATL.  Figure 8 displays the ability to track an entire nephron with manual intervention.

The number of manual corrections varies with the sensitivity of the validation steps. For example, decreasing the ANN threshold, increasing the coefficient of distance validation, or turning bidirectional validation off will decrease the number of requests for manual correction by the algorithm. However, this increases the chance of tracking incorrect structures (decreases *α*) as shown conceptually in Figure 5. The settings of the validation steps were therefore chosen such that the algorithm tracks with high accuracy (*α*) while not requesting excessive unnecessary manual interventions.

### 9.2. Efficacy of Validation Steps

The validation steps for a particular move are carried out in a set sequence with the least computationally expensive step being first. This is so that if an invalid move is detected, it does not have to go through all of the subsequent stages. However, for testing, all validation steps were carried out.

#### 9.2.1. Validation through the Rule Base

Although the types of invalid moves are diverse, the rule base attempts to model the majority through hard-coded, direct rules while the ML validation attempts to model them in a more generalised, less rigid manner. The rejection rates and accuracies are detailed in Table 3.

All four rules produce accuracies above 90% with the distance validation rule being the most accurate (99.67%) and the machine learning validation being the most often triggered (captures 57.61% of all invalid moves). Given a large set of detected invalid moves, certain fractions are uniquely captured by each of the validation steps as shown in Table 3. Of the 8017 invalid moves, 49.65% were measured as being captured by more than one rule.

Ideally, the ML validation stage should be able to perform the tasks of distance and skipping validation, as the rules should be spontaneously integrated into the learnt hypothesis. Since 57.54% of the moves captured by the machine learning step are captured by other rules, it can be said that it does perform the tasks of the rule base to some degree. It can also be said that the rule base models the abnormalities to a good degree since the majority of invalid moves are eliminated even without the machine learning component.

#### 9.2.2. Validation through an ANN and SVM

The machine learning algorithms eliminate a large number of invalid moves which would have otherwise resulted in multiple nephrons, interstitial tissue, and blood vessels being linked (42.46% of detections are unique). The labelled dataset consisted of 9424 examples, which was split into training, validation, and test sets with a 0.7 : 0.15 : 0.15 ratio, respectively.

Both the ANN and SVM produced a classification accuracy of approximately 93% on the test set, with the ANN being purposely less sensitive (84% for the ANN compared to 90% for the SVM) in order to minimise the number of false positives. The confusion matrix and performances are detailed in Table 4.

The impact of different features on classifying different types of examples is visualised and deduced using Principal Component Analysis (PCA), a dimensionality reduction technique. PCA of the features revealed that the shape profile feature is most significant when differentiating between classes 1 and 2, while shape factors play more of a role in distinguishing classes 3 and 4.

### 9.3. Processing Times

The current implementation is not optimally efficient, although the main aim was to develop the technique rather than optimising efficiency for an end-user application. Computational bottlenecks include the discrete Fourier transform required for image alignment, continuous calling of the ANN structure, and reading in three images per iteration of the algorithm. An implementation of the system using C++ or another more efficient language would decrease execution time. Parallel processing and use of a graphics processing unit for imaging operations would also improve speed.

## 10. Analysis and Discussion

The validation steps generally increase accuracy (*α*) while manual intervention increases the extent to which a nephron is tracked (*β*). Each portion of the nephron is discussed with reference to the results in Table 2. A result applies to both the mouse and rat datasets if it is not explicitly distinguished.

From the measured *β* values, up to 43% of a nephron's length is made up of the PCT and PST. The algorithm is able to track the full length of the PCT and PST with 1–3 and 2–15 manual corrections in the mouse and rat, respectively, when large distortions and artefacts are detected.

Although the PCT was predicted to be the most challenging part of the nephron to track due to its convoluted nature, it is tracked with high accuracy (*α* = 95.14% in the mouse and *α* = 96.34% in the rat) as follows.

- The cross sections are well isolated as they are large in diameter (15–30 pixels wide) and well defined (they have thick walls).
- The average distance between neighbouring cross sections (≈25 pixels) is larger than the average image misalignment of 4 pixels.

Similarly, the PST of the mouse is tracked well with *α* = 98.24% as the cross sections are well isolated and defined and the paths have a relatively straight course. In comparison, tracking of the rat PST produced a lower accuracy of 90.17% due to a higher frequency of tissue folds leading to incorrect linking with other nephrons.

A class 2 move is successfully detected by the ML algorithms when the PCT of a nephron joins the glomerulus at its urinary pole, thus terminating the tracking. Without this, fragments in the glomerulus would be tracked towards the vascular pole, and tracking would continue through the adjoining afferent/efferent arteriole, which then joins blood vessel systems and other glomeruli, which is undesirable. When the PST narrows into the DTL, a class 5 move is successfully triggered. The level of the class 5 output is used as a region signal to change the mode of tracking into a unidirectional one for the inner medulla. This reduces error in tracking in the inner medulla tremendously as ambiguity decreases when only one unidirectional path is allowed to be formed.

The DTL in mouse and rat kidneys is tracked with only moderate accuracies of *α* = 80.57% and *α* = 84.63%, respectively, as the cross sections are very small in diameter (3–8 pixels) and very dense (≈6 pixels between neighbouring cross sections). This results in a higher error probability during tracking as these values are comparable to the average misalignment of 4 pixels. Confusion is more likely among identical, closely packed nephrons which are not ideally aligned. The DTL requires many manual corrections (27 on average in the rat) to produce a high *β* value. Frequent premature termination occurs because the cross sections are less well defined, making it more difficult to isolate them (very thin nephron walls cause independent cross sections to merge in the binary image), which results in missing cross sections and invalid moves as seen by the ANN.

The ATL faces the same challenges as the DTL. However, these cross sections are slightly larger (6–12 pixels) and have thicker walls and are thus tracked more accurately in comparison to the DTL. The ATL requires about half the number of manual corrections when compared to the DTL in both the mouse and rat datasets.

The TAL is tracked well (with 96.32% and 97.48% accuracies in the mouse and rat, resp.) as its cross sections are well isolated and relatively large (8–12 pixels in the mouse and 13–20 pixels in the rat), and the path is straight.

The DCT differs vastly in the mouse and rat datasets. In the mouse, the DCT remains narrow as it progresses from the TAL. The small cross sections making up a convoluted path are difficult to track. Fast changes in morphology (due to only having every second slice) combined with small-sized cross sections trigger the distance validation rule. An average of 5 corrections is required in the mouse DCT.

The rat DCT is tracked well as its characteristics are comparable to the rat PCT. The cross sections are much larger than in the mouse. Although the DCT is longer in the rat, it also requires an average of 5 corrections. Branching is correctly handled when the DCT of multiple nephrons join through a common collecting duct.

Manual intervention is useful when the path terminates prematurely (usually due to image defects), as the user simply bypasses the problematic cross section. In cases where incorrect links are made between different nephrons, manual intervention is not useful. The latter case is difficult to identify and correct without comparison to the manually tracked data or by manual inspection.

In general, the results are highly dependent on the quality of the images, the size of the nephron cross sections, and the amount of interfering interstitial tissue. Thicker slices (e.g., every second slice in the mouse (5 *μ*m) compared to every slice in the rat (2.5 *μ*m)) also produce less accurate results as the change in morphology is then more abrupt from image to image. Local image distortions and low image resolution in images of the inner medulla are the main limiting factor in automatically tracking full nephron paths.

A high frequency of images containing artefacts and tissue folds decreases the accuracy of the findings tremendously, as it only requires a single incorrect move to cause the path to deviate from the nephron at hand onto another structure (i.e., the stability of the tracking process is completely dependent on the results of the current iteration). This is especially applicable for tracking in the inner medulla, where high tubule density coupled with an artefact may result in two nephron cross sections joining incorrectly and the turn being mistaken for a loop of Henle.

## 11. Future Work

Further studies would be required to establish if the method developed is sufficiently generic to be used to map the architecture of other anatomical structures such as blood vessel networks in tomographic CT and MRI images. The learning algorithm would require retraining on new examples, and parameters could be tuned to control algorithm sensitivity, allowing the system to adapt to the features of different structures. The applicability and adaptability of this system to other fields are an avenue for future work.

### 11.1. Recommendations for Future Histological Image Sets

Higher resolution images would offer improved accuracy in isolation and tracking of cross sections in the inner medulla. Another useful addition would be using markers on the slides to aid automatic image alignment, as well as eliminating or marking highly distorted images.

A previous study by Pannabecker and Dantzler [2, 3] manually reconstructed rat nephrons using immunohistochemically stained sections (antibodies which bind to segment specific proteins) to stain various parts of the nephrons. This resulted in the DTL, ATL, collecting duct, and blood vessels fluorescing with different colours. Such staining methods would provide differentiating colour information and features to the tracking and machine learning algorithms, respectively. The confidence of results would increase as different types of cross sections could easily be distinguished from one another and interstitial tissue interference would be virtually eliminated as only cross sections of interest would be highlighted. A drawback is that the morphology of the tubules may not be intact as only particular features of the tubules would be stained.

## 12. Conclusion

The aim of the present study was to develop an automated system for the tracking of nephrons. A proposed methodology involving image processing and a custom tracking algorithm supervised by machine learning algorithms is presented. A number of features are extracted in order to retain shape information during the data abstraction process. The ANN and SVM have high classification accuracies and eliminate invalid moves caused by a number of hindering factors such as artefacts. The presented system is able to successfully track large portions of the nephrons automatically through both highly convoluted and straight paths. Particularly, the PCT, PST, and TAL are tracked with >90% accuracies in the mouse and rat datasets and form more than half of the nephron length. While only portions of the paths can be obtained automatically from the starting seed, full nephron paths can be obtained with an average of 17 and 62 manual corrections in the mouse and rat datasets, respectively. This is reasonable considering the thousands of coordinates making up each nephron path. Although complete automation is still elusive, the system saves a considerable amount of time and effort compared to the manual tracking task as it performs 99% of the task automatically. Performance may improve with further training of the machine learning algorithms, optimising automatic parameter variation, and manually eliminating image artefacts. The methods developed during this study form a foundation for further development towards a fully automated nephron tracking system.

## Figures and Tables

**Table 1:** The intermediate output classes of the learning functions and their combination into final classes.

Final class	Intermediate class
Valid move	(1) A normal move between circular cross sections
	(2) A normal move involving elongated cross sections
Invalid move	(3) An abnormal move typically involving interstitial tissue or blood vessel cross sections
	(4) A move involving a glomerulus cross section
x	(5) A move in the inner medulla

**Table 2:** Test results on a chosen set of 16 mouse nephrons and 11 rat nephrons. The number of manual corrections is given as the mean ± one standard deviation. Ideal *β* values for the six segments for both the mouse and rat were derived from measurement of manual data and the results in the appendix of the previous study [8].

Area of nephron	*β*_IDEAL_ (%) [8]	Mouse				Rat			
		*β*_MEAN_ (%)	Extent: *β*_MEAN_/*β*_IDEAL_ (%)	Accuracy: *α*_MEAN_ (%)	Average number of manual corrections	*β*_MEAN_ (%)	Extent: *β*_MEAN_/*β*_IDEAL_ (%)	Accuracy: *α*_MEAN_ (%)	Average number of manual corrections
PCT	25	27.36	109.44	95.14	1.20 ± 1.11	28.48	113.92	96.32	5.20 ± 4.70
PST	18	16.33	90.72	98.24	0.50 ± 0.71	14.64	81.33	90.17	5.00 ± 2.75
DTL	19	13.90	73.16	80.57	5.44 ± 1.69	15.83	83.32	84.63	24.00 ± 8.19
ATL	14	14.94	106.71	85.67	2.46 ± 1.87	15.63	111.64	88.47	13.50 ± 6.95
TAL	14	13.19	94.21	96.32	3.64 ± 1.55	11.50	82.14	97.48	6.67 ± 3.09
DCT	10	14.29	142.90	72.13	5.86 ± 3.00	13.91	139.10	95.23	4.33 ± 2.49
Full	100	100	100	87.49	19.09 ± 1.65	100	100	80.85	58.70 ± 4.70

**Table 3:** The invalid move rejection rate and accuracies of the validation steps are shown. Results are based on 8017 invalid moves.

Validation step	% of total invalid moves flagged	% of detected invalid moves that are unique	% accuracy
Distance Val.	40.21	25.94	99.67
Skip Val.			
Total	38.59	25.38	90.01
Skips	98.97		
Bidirec. Val.	29.92	18.94	92.05
ML Val.	57.61	42.46	93.62

**Table 4:** Results of the ANN and SVM on the test set of 712 examples. The 5 classes have been condensed into valid and invalid classes for final classification.

Classification algorithm	Predicted class	Target class		Performance indicators (%)		
		Valid	Invalid	Accuracy	Precision	Sensitivity
ANN (threshold = 0.3)	Valid	492	32	93.82	93.62	84.61
	Invalid	12	176			

SVM with RBF kernel (width = 5)	Valid	475	19	93.25	86.70	90.86
	Invalid	29	189			
